# Retroperitoneal Dedifferentiated Liposarcoma With Penetrating Abscess Formation Secondary to Ileal Invasion: A Case Report

**DOI:** 10.1002/ccr3.71561

**Published:** 2026-01-07

**Authors:** Kenichiro Yambe, Kei Nakagawa, Kuniharu Yamamoto, Hiroto Sakurai, Kazuhiro Takami, Noriko Kondo, Chikashi Shibata, Yu Katayose

**Affiliations:** ^1^ Department of Hepato‐Biliary‐Pancreatic Surgery Tohoku Medical and Pharmaceutical University Hospital Sendai Japan; ^2^ Department of Gastrointestinal Surgery Tohoku Medical and Pharmaceutical University Hospital Sendai Japan

**Keywords:** abscess, dedifferentiated liposarcoma, enterocutaneous fistula, intestinal penetration, MDM2 amplification, retroperitoneal tumor

## Abstract

Liposarcoma is a relatively common malignant soft tissue tumor in adults. Dedifferentiated liposarcoma (DLS) is a particularly invasive subtype characterized by high recurrence rates and a poor prognosis. We describe a case of a retroperitoneal liposarcoma with poor differentiation that presented with peritonitis accompanied by ileal infiltration and perforating abscess formation. A 77‐year‐old male presented to our hospital with abdominal pain and fever. Computed tomography revealed a 13 cm tumor in the right lower abdomen with internal air density. An ileocecal resection was performed. Histopathological examination revealed a solid proliferation of spindle‐shaped atypical cells. Immunohistochemical results were positive for MDM2 and negative for CD34 and c‐kit. Fluorescence in situ hybridization confirmed MDM2 amplification, leading to a diagnosis of dedifferentiated liposarcoma. The postoperative course was initially favorable; however, 6 months later, a penetrating abscess recurred and progressed to an enterocutaneous fistula. Intestinal penetration by dedifferentiated liposarcomas is extremely rare. This case highlights the challenges of preoperative diagnosis, the importance of surgical strategies that balance infection control with curative resection, and the need for multidisciplinary management of recurrence.

## Key Clinical Message

Dedifferentiated liposarcomas with intestinal invasion and abscess formation are rare. Preoperative pathological diagnosis remains challenging, requiring biopsy confirmation. When complete resection is unfeasible, prioritizing diagnosis and complication avoidance over incomplete resection enables a smooth transition to postoperative multidisciplinary treatment, preserving patient activities of daily living.

**Abbreviations**
CDK4: cyclin‐dependent kinase 4
CT: computed tomography
DDLPS: dedifferentiated liposarcoma
FISH: fluorescence in situ hybridization
GIST: gastrointestinal stromal tumors
MDM2: mouse double minute 2 homolog
MRI: magnetic resonance imaging
PT: prothrombin time
QOL: quality of life
WDLPS: well‐differentiated liposarcoma

## 1 Introduction

Liposarcoma accounts for approximately 15%–20% of malignant soft tissue tumors in adults, making it relatively common [1]. According to the World Health Organization, liposarcomas can be categorized into well‐differentiated, dedifferentiated, myxoid, pleomorphic, and mixed types [2]. Among these, dedifferentiated liposarcoma (DDLPS) represents 10%–20% of all liposarcomas [3]. DDLPS commonly arises in the retroperitoneum and is characterized by high recurrence rates and local invasiveness, making it a subtype with a poor prognosis [4].

The incidence of retroperitoneal liposarcoma is reported to be 2.5–2.7 cases per million people, with approximately 30% classified as the dedifferentiated type [5, 6]. Among retroperitoneal tumors, liposarcomas account for approximately 12%–40% [7, 8].

DDLPS exhibits greater cellular atypia than well‐differentiated liposarcoma (WDLPS) and presents with distinct histological dedifferentiation. These tumors are often already massive at the time of diagnosis, with many cases being unresectable or demonstrating invasion of neighboring organs. Macroscopically, they frequently contain areas devoid of fatty components, which complicates their identification as tumorous lesions on imaging [4].

Histologically, DDLPS is considered to arise from WDLPS, with overexpression of mouse double minute 2 homolog (MDM2) and cyclin‐dependent kinase 4 (CDK4), which serve as characteristic markers [9]. It predominantly affects middle‐aged to elderly adults (50–70 years), with a slight male predominance and a median patient age of 61–67 years [7]. Approximately 90% of the cases occur as primary tumors, whereas secondary dedifferentiation from well‐differentiated tumors accounts for the remaining 10% [5].

Contrast‐enhanced computed tomography (CT) and magnetic resonance imaging (MRI) are widely used for the diagnosis; however, imaging findings alone do not allow for a definitive diagnosis of DDLPS, making histopathological examination essential. MDM2 gene amplification is a highly specific marker of DDLPS, and confirmation via immunohistochemistry or fluorescence in situ hybridization (FISH) enables its differentiation from other liposarcoma subtypes [10]. In particular, immunohistochemistry combined with CDK4 is a highly sensitive and specific diagnostic method. Recent reports have indicated that combining p16 immunostaining with MDM2 and CDK4 improves the accuracy of the differential diagnosis of atypical lipomatous tumors/WDLPS and DDLPS.

Complete resection (R0) is the first‐line treatment, and en bloc resection is recommended whenever feasible [11]. However, recurrence rates remain high, with 5‐year recurrence rates of 30%–50% [12], often necessitating reoperation or chemotherapy.

Regarding chemotherapy, anthracycline‐based agents, such as doxorubicin, are commonly used [13]; however, therapeutic responses are limited and require careful case selection for effectiveness.

Retroperitoneal liposarcomas frequently reach mean sizes of 20–30 cm at the time of diagnosis, with some considered “giant” (> 30 cm) [14]. A distinctive characteristic of these tumors is the late onset of symptoms, which typically appear only when the tumor invades adjacent organs, occasionally leading to severe complications such as intestinal obstruction, penetration, or abscess formation. Cases of liposarcomas penetrating the intestine and forming an abscess are extremely rare, with only a few reported cases worldwide (Table 1).

**TABLE 1:** Reported cases of liposarcomas with digestive tract invasion.

Author (year)	Age/Sex	Primary site	Size (cm)	Invaded organ	Penetration/abscess	Treatment	Outcome
Johnson et al. (2010)	65/M	Retroperitoneum	18	Colon	Yes/No	En bloc resection	NED 6 mo
Garcia et al. (2012)	72/F	Retroperitoneum	24	Jejunum	Yes/Yes	Partial resection	DOD 8 mo
Kim et al. (2014)	58/M	Retroperitoneum	15	Duodenum	No/No	En bloc resection	NED 12 mo
Lee et al. (2015)	63/M	Mesentery	12	Ileum	No/No	En bloc resection	NED 24 mo
Wang et al. (2016)	59/F	Retroperitoneum	20	Colon	No/No	En bloc resection	AWD 18 mo
Smith et al. (2018)	68/M	Retroperitoneum	22	Stomach	No/No	Partial resection	DOD 6 mo
Brown et al. (2019)	54/F	Retroperitoneum	26	Jejunum	Yes/Yes	En bloc resection	AWD 14 mo
Chen et al. (2020)	74/M	Retroperitoneum	17	Ileum	No/No	En bloc resection	NED 9 mo
Wilson et al. (2022)	66/F	Mesentery	14	Ileum	No/No	En bloc resection	NED 36 mo
Present case (2025)	77/M	Retroperitoneum	13	Ileum	Yes/Yes	Partial resection	AWD 7 mo

Abbreviations: AWD, alive with disease; DOD, died of disease; mo, months; NED, no evidence of disease.

Although our patient's tumor measured 13 cm and did not meet the criteria for a giant tumor, it exhibited unusually aggressive local complications: ileal invasion, perforating abscess formation, and eventual progression to an enterocutaneous fistula upon recurrence. This exceptionally rare clinical course presents several critical clinical implications, including the difficulty of accurate preoperative diagnosis, the challenge of determining the appropriate extent of resection in an emergency setting, and the complexities of recurrence management. Here, we describe the case and review the literature to provide valuable insights into surgical limitations, the feasibility of conservative management, and the crucial role of multidisciplinary strategies for DDLPS when complete resection is not achievable.

## 2 Case History/Examination

The patient was a 77‐year‐old male who experienced lower abdominal pain and fever 2 days before presentation and was referred to the general/internal medicine department of our hospital via a local clinic. His medical history included hypertension, rheumatoid arthritis, and chronic renal failure secondary to nephrosclerosis. Medications administered included prednisolone (6 mg/day), salazosulfapyridine, celecoxib, rebamipide, esomeprazole, spironolactone, febuxostat, pitavastatin, diltiazem, and valsartan.

On admission, vital signs were as follows: temperature, 37.9°C; blood pressure, 134/78 mmHg; pulse, 84 beats/min; and SpO_2_, 98% (room air). The patient was alert and oriented with no apparent abnormalities in the heart or lungs. Abdominal examination revealed tenderness in the lower abdomen, but no rebound tenderness or muscular defense. Bowel sounds were within the normal range. Lower limb edema, skin abnormalities, or lymphadenopathy were not observed.

Laboratory findings revealed elevated inflammatory markers, with a white blood cell count of 10,300/μL and a C‐reactive protein level of 5.67 mg/dL. Red blood cell count was 4.15 × 10^6^/μL, hemoglobin 13.6 g/dL, hematocrit 40.6%, and platelet count 18.5 × 10^4^/μL. Liver and biliary enzyme levels were within normal limits: aspartate aminotransferase, 15 U/L; alanine aminotransferase, 16 U/L; lactate dehydrogenase, 173 U/L; alkaline phosphatase, 67 U/L; gamma‐glutamyl transpeptidase, 48 U/L; and total bilirubin, 0.6 mg/dL. Renal function was mildly decreased, with blood urea nitrogen 29 mg/dL, creatinine 1.29 mg/dL, and estimated glomerular filtration rate 42.4 mL/min/1.73 m^2^. Coagulation parameters were within the normal ranges, with a prothrombin time (PT) of 90% and a PT‐international normalized ratio of 1.07. Arterial blood gas (room air) showed a pH of 7.384, partial pressure of carbon dioxide 39.8 mmHg, partial pressure of oxygen 110.0 mmHg, bicarbonate 23.7 mmol/L, standard base excess −1.3 mmol/L, with an elevated lactate level of 3.1 mmol/L.

**FIGURE 1:**
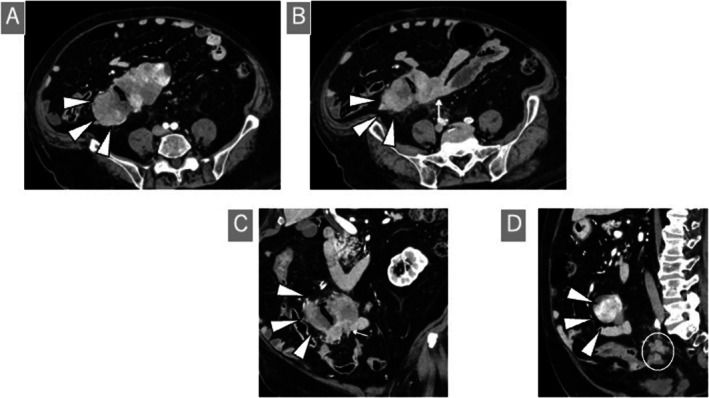
Preoperative contrast‐enhanced computed tomography (CT). Abdominal contrast‐enhanced CT tomography showing a 13 cm tumor in the right lower abdomen. (A–C) Air density within the tumor suggests intestinal penetration with abscess formation. The lesion extended from the terminal ileum to the mesentery and retroperitoneum, displacing the adjacent small intestine. (B, C) Suspected disseminated nodules are visible anterior to the sacrum. (D) Axial view showing the relationship between the tumor and the surrounding structures.

Contrast‐enhanced abdominal CT revealed a 13 cm tumor in the right lower abdomen. The tumor extended from the dorsal ileal mesentery to the terminal ileum, primarily occupying the retroperitoneal space with indistinct borders and mixed fat and soft tissue densities. The presence of internal air suggests intestinal penetration and abscess formation. The tumor was in contact with the horizontal portion of the duodenum and the right ureter, causing displacement of the surrounding small intestine. No apparent strictures or dilations were observed in the small intestines. The tumor was located dorsal to the mesentery of the small intestine, suggesting a retroperitoneal primary tumor with intestinal invasion. Suspected disseminated nodules are observed anterior to the sacrum (Figure 1).

## 3 Differential Diagnosis

The differential diagnosis included several possibilities based on the imaging findings and clinical presentation.

Although preoperative imaging primarily suggested a retroperitoneal tumor, other possibilities included mesenteric or perforated small intestinal tumors. However, qualitative determination is difficult without a pathological examination. Approximately 12%–40% of retroperitoneal tumors are liposarcomas, with a high prevalence of DDLPS6–8. Other representative retroperitoneal tumors include leiomyosarcomas, malignant peripheral nerve sheath tumors, and epithelioid sarcomas. In contrast, mesenteric tumors are rare. Gastrointestinal stromal tumors (GIST) and lymphomas are among the main differential diagnoses, although they tend to be relatively localized and are less likely to exhibit extensive invasion.

Thus, while retroperitoneal liposarcoma was strongly suspected, mesenteric tumors and primary intestinal tumors also required consideration.

## 4 Outcome and Follow‐Up

An emergency ileocecal resection was performed.

**FIGURE 2:**
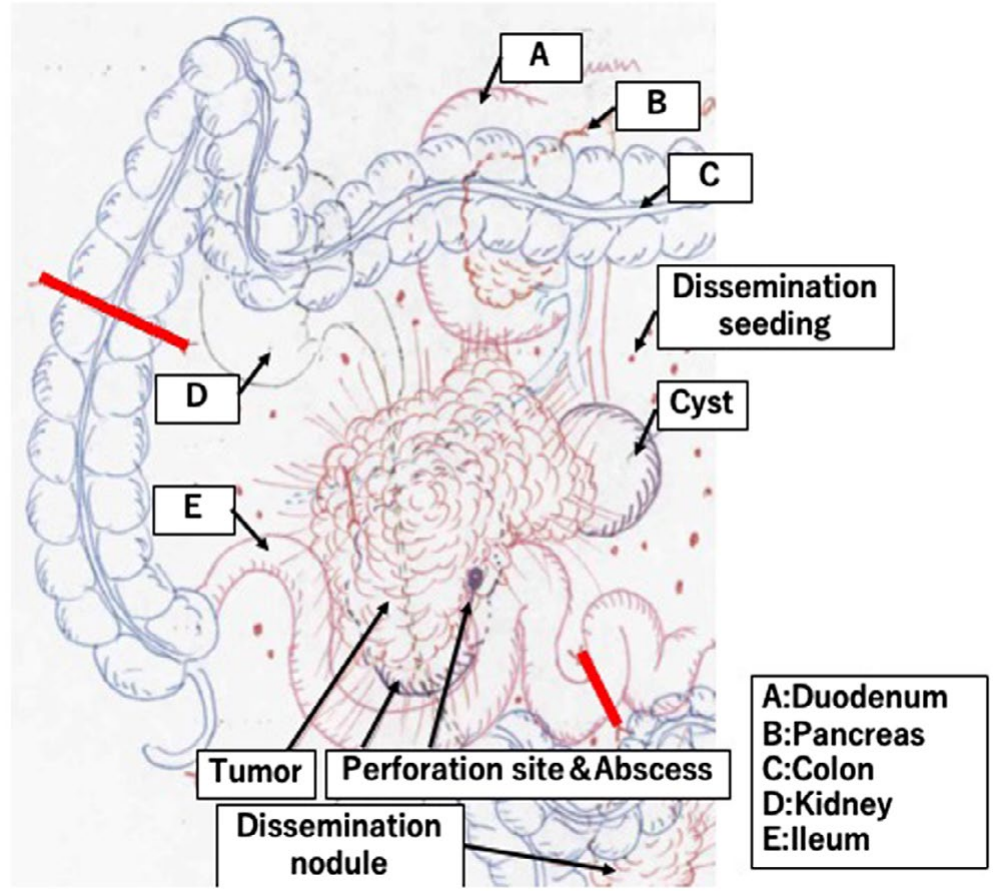
Intraoperative findings. The tumor invaded the horizontal portion of the duodenum and right ureter, extending from the terminal ileum to the retroperitoneum. Multiple disseminated nodules were observed in the mesentery and anterior sacrum. (A) Duodenum, (B) pancreas, (C) colon, (D) kidney, (E) ileum.

Intraoperative findings revealed a tumor dorsal to the ileal mesentery and 10 cm oral to the ileocecal valve, with invasion into the duodenum and right ureter, as well as disseminated white nodules from the mesentery to the anterior sacral surface. Because R0 resection was deemed unfeasible, priority was given to controlling the infection source by resecting the penetrated ileocecal region. R0 resection was impossible because the tumor invaded the duodenum, the right ureter, and the sacral surface, and disseminated lesions were present, making en bloc resection infeasible. The invaded areas of the duodenum and ureter were preserved (Figure 2). The operative time was 237 min, with blood loss of 597 mL.

**FIGURE 3:**
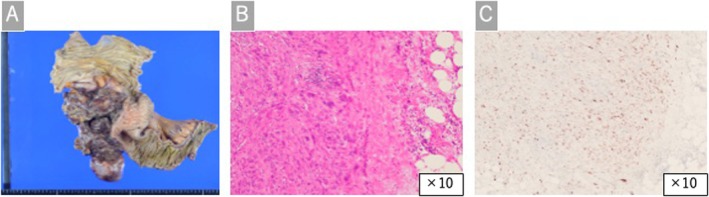
Histopathological findings. (A) Macroscopic view showing a 13 cm tumor invading from the serosal side to the ileal mesentery, with areas of hemorrhage and necrosis. (B) Hematoxylin and eosin staining showing solid proliferation of spindle‐shaped atypical cells with necrosis and significant nuclear atypia. (C) Immunohistochemical analysis was positive for mouse double minute 2 homolog, alpha‐smooth muscle actin, vimentin, and HHF35, whereas negative for CD34, c‐kit, DOG‐1, cytokeratin, desmin, S100, MyoD1, and cyclin‐dependent kinase 4.

The postoperative course was favorable, with oral intake resuming on the third postoperative day and discharge on the twelfth day. Histopathological examination revealed a white tumor invading the ileal wall, accompanied by hemorrhage, necrosis, and abscess cavity formation. Spindle‐shaped atypical cells proliferated irregularly and displayed nuclear atypia and numerous mitotic figures. Immunohistochemical analysis was positive for MDM2, vimentin, α‐smooth muscle actin, and HHF35, whereas negative for CD34, c‐kit, DOG‐1, cytokeratin, desmin, S100, MyoD1, and CDK4. FISH confirmed MDM2 gene amplification, leading to a final diagnosis of DDLPS (Figure 3).

Chemotherapy was proposed during the outpatient follow‐up; however, the patient declined regular monitoring. Six months postoperatively, the patient experienced a recurrence of abdominal pain and fever, and CT confirmed abscess recurrence with small intestinal penetration. Although a temporary improvement was achieved with antibiotic therapy and fasting, an enterocutaneous fistula developed 7 months later. Fortunately, the abscess and intestinal fluid did not spread into the peritoneal cavity, and effective drainage through the fistula allowed for continued home care with pouch management.

## 5 Discussion

To date, only two reported cases have described DDLPS with digestive tract penetration and associated abscess formation, and only 10 cases have reported digestive tract invasion [15, 16, 17, 18, 19, 20, 21, 22, 23, 24]. Abscess formation has rarely been documented. However, emergency surgery was performed in both previously reported perforation cases [15, 16, 17, 18, 19, 20, 21, 22, 23, 24] (Table 1).

For a definitive diagnosis, MDM2 amplification is useful in addition to histological evaluation [6, 7, 8]. MDM2 positivity on immunohistochemistry is specific to DDLPS and aids in differentiating it from other sarcomas and GISTs. MDM2 amplification by FISH has high sensitivity and specificity, making it an essential diagnostic tool, particularly when fatty tissue components are scarce [9]. In the present case, both MDM2 positivity and gene amplification confirmed the diagnosis.

In the treatment selection for DDLPS, R0 resection is the primary goal when feasible [11]. The latest National Comprehensive Cancer Network guidelines recommend en bloc resection to achieve local control of retroperitoneal liposarcomas. Specifically, even when a tumor invades the intestines, kidneys, pancreatic tail, or spleen, surgery with combined resection should be performed if R0 resection is possible [25, 26]. However, in practice, retroperitoneal tumors are often already massive at diagnosis, with invasion into multiple organs, making aggressive combined resection difficult because of functional issues and deterioration of the patient's general condition [4]. Large‐scale multi‐institutional reviews demonstrate that multivisceral resection is required in a substantial proportion of retroperitoneal liposarcoma surgeries. The Trans‐Atlantic RPS Working Group reported that contiguous organ resection was performed in approximately 70%–80% of primary operations, with kidney resection required in about 55% and bowel resection (colon/rectum) in approximately 57% of cases. These findings highlight how frequently organ and intestinal resection is necessary when attempting R0 resection in retroperitoneal DDLPS [27, 28].

In the present case, the ileocecum, including the area with the penetrating abscess, was resected while preserving the invading duodenum and ureter. This approach was based on the intraoperative findings of multiple disseminated lesions, which rendered curative resection (R0 resection) infeasible [11] and the determination that resection of the penetrated area was necessary to improve the patient's general condition. Although alternative management could have included stoma creation and antibiotic therapy for penetrating the intestinal abscess, followed by tumor biopsy for histological diagnosis and consideration of multimodal treatments combining pharmacotherapy and surgical resection [29], previous reports indicate that all patients with liposarcoma involving digestive tract penetration and abscess formation underwent surgical resection, including the penetrated abscess area, with no reports of successful control through conservative treatment or palliative surgery.

Additionally, the invasion of DDLPS may be suspected based on preoperative imaging, primarily when a retroperitoneal primary tumor is considered. However, retroperitoneal tumors are difficult to diagnose because of their size and structural diversity and often lack clear imaging features indicative of liposarcoma. In particular, when dedifferentiated components are predominant, the proportion of fat density is low, complicating an accurate diagnosis, even with fat suppression on MRI T1‐weighted images or diffusion‐weighted imaging [29].

Regarding prognosis, DDLPS exhibits a high local recurrence rate, reaching 41%–52% at 5 years post‐surgery for retroperitoneal primary cases [11]. However, the distant metastasis rate is relatively low (approximately 15%–17% [12], and the disease‐specific mortality rate is high, 28%–30%). The frequency of distant metastasis is lower than that of other high‐grade sarcomas. However, once metastasis occurs, the prognosis is poor, with a median survival time of approximately 5 months after diagnosis [30].

Doxorubicin monotherapy or combination therapy with ifosfamide has traditionally served as the standard treatment for soft tissue sarcomas [31]. Doxorubicin, in particular, has a reported response rate of 15%–25%, although responsiveness varies greatly depending on the tumor subtype. In DDLPS, some patients demonstrate a therapeutic response, whereas others show minimal tumor reduction or poor disease control, resulting in a low overall response rate. Recently, molecular targeted therapies, such as MDM2 inhibitors (e.g., milademetan and bringimadlin), have been investigated; however, their clinical use remains limited and has not yet been established as a standard treatment. Phase II trials in Europe and the United States demonstrated partial responses; however, challenges persist in managing adverse events and their long‐term effects [29, 31].

Based on these literature findings, we examine the clinical implications of this case. The retrospective analysis of this case highlights several critical considerations for treatment strategies. This was a case of gastrointestinal perforation, abscess, and localized peritonitis in an elderly patient in whom rapid diagnosis and surgical intervention were considered necessary. However, owing to disseminated nodules and multiorgan invasion rendering complete resection unfeasible, cytoreductive surgery, including resection of the penetration site, was performed. However, residual tumor growth leads to the formation of new penetration sites, ultimately resulting in decreased quality of life (QOL). In cases requiring emergency surgery, resection of the penetration site is often performed to control the infection. However, tissue biopsy and stoma creation may have facilitated a definitive diagnosis and informed subsequent treatment decisions. Currently, novel therapies such as MDM2 and immune checkpoint inhibitors are being evaluated in clinical trials and may potentially complement cytoreductive surgery. Residual penetration sites may interfere with postoperative pharmacotherapy, for instance, by causing treatment interruptions due to infection. Further reports on the outcomes of stoma creation alone without resection of the penetration site are awaited. In this case, the patient's refusal to undergo pharmacotherapy led to residual tumor progression, contributing to long‐term functional decline and challenges in maintaining activities of daily living (ADL). This underscores the importance of patient counseling regarding the necessity of multimodal treatment in advanced cases. Rapid tissue diagnosis and early integration of emerging therapies such as MDM2 inhibitors are critical in shaping treatment strategies for cases where complete resection is not feasible.

In conclusion, this is an extremely rare case of DDLPS with ileal invasion and penetration, abscess formation, and eventual progression to a cutaneous fistula. Preoperative qualitative diagnosis was difficult, and confirmation of MDM2 gene amplification in the resected specimen was essential for a definitive diagnosis. Given the difficulty in achieving R0 resection, palliative resection was performed; however, the residual tumor enlarged postoperatively, causing re‐penetration and enterocutaneous fistula formation, complicating long‐term quality of life (QOL) maintenance. Therefore, we must propose what can be done before deciding on tumor resection.

In future cases, when extensive tumors are identified in the retroperitoneum or mesentery on imaging studies, DDLPS and other lesions should be considered as differential diagnoses. If the tumor cannot be resected as a single mass, options such as creating an ileostomy and controlling peritoneal inflammation by preserving the biopsy site or draining the perforation site should be considered. When complete resection is challenging, early introduction of multimodal treatment, including emerging molecular targeted therapies, such as MDM2 inhibitors, should be considered. This case has educational significance as it highlights the importance of such strategies. Even if a definitive diagnosis of DDLPS is not established, once the possibility of an unresectable malignant disease is considered, it is important not to forcibly resect the site of penetration. Furthermore, establishing strict follow‐up protocols based on high recurrence risk and enabling a rapid response to recurrence may contribute to maintaining long‐term QOL.

This case report demonstrates the challenges and strategies in diagnosing and treating DDLPS with rare complications, providing valuable insights for managing similar cases in the future.

## Author Contributions

**Kenichiro Yambe:** writing – original draft. **Kei Nakagawa:** project administration, writing – review and editing. **Kuniharu Yamamoto:** writing – review and editing. **Hiroto Sakurai:** writing – review and editing. **Kazuhiro Takami:** writing – review and editing. **Noriko Kondo:** writing – review and editing. **Chikashi Shibata:** writing – review and editing. **Yu Katayose:** writing – review and editing.

## Funding

The authors have nothing to report.

## Ethics Statement

This case report was conducted following the ethical standards of the Institutional and National Research Committee and the 1964 Declaration of Helsinki and its later amendments. Research Ethics Committee for Life Science and Medical Research, Tohoku Medical and Pharmaceutical University (No. 2025‐4‐023‐0000).

## Consent

Written informed consent was obtained from the patient for the publication of this case report and the accompanying images.

## Conflicts of Interest

The authors declare no conflicts of interest.

## Data Availability

Data sharing not applicable to this article as no datasets were generated or analyzed during the current study.
